# Elements of Weight Management Among Pre-Kidney Transplant Candidates: The Patient Perspective

**DOI:** 10.3389/ti.2024.12735

**Published:** 2024-05-24

**Authors:** Ariana Chirban, Diana D. del Valle, Taylor Coe, Maria P. Cote, Maggie Chen, Jennie Cataldo, Nahel Elias, Anushi Shah, Leigh Anne Dageforde

**Affiliations:** ^1^ San Diego School of Medicine, University of California, San Diego, CA, United States; ^2^ Department of Surgery, Division of Transplantation, Massachusetts General Hospital, Boston, MA, United States; ^3^ Harvard Medical School, Boston, MA, United States; ^4^ Beth Israel Deaconess Medical Center, Boston, MA, United States; ^5^ School of Medicine, University of Maryland, Baltimore, MD, United States

**Keywords:** kidney transplant, waiting list, obesity, weight perception, weight loss

## Abstract

Obesity and related comorbidities heighten risks for complications in kidney transplant settings. While pre-transplant patients often have access to nutrition counseling and health support, literature is limited on patients' perceptions of weight and motivation to lose weight prior to transplantation. We conducted a survey among ≥18-year-old patients on the kidney transplant waitlist at a single center. Questions addressed weight perception, motivation for weight loss, available resources, and engagement in physical activity. Medical records provided demographic and clinical data. Statistical tests analyzed quantitative data, while free-text responses were thematically grouped and described. Of 1055 patients, 291 responded and were matched with demographic data. Perceived weight changes correlated with actual changes in body mass index (BMI) (<24.9) were more receptive to weight center resources (<30 kg/m^2^) are most interested in weight loss resources and demonstrate motivation. Furthermore, pre-transplant nutrition counseling correlates with healthier behaviors. Integrating patients’ perspectives enhances pre-transplant protocols by encouraging active involvement in health decisions.

## Graphical Abstract

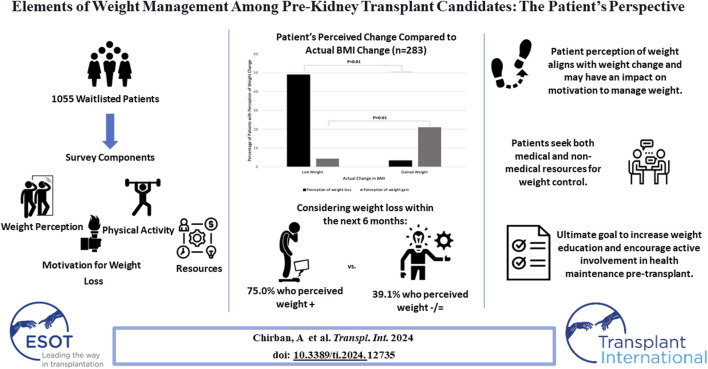

## Introduction

Obesity in the U.S. has been increasing steadily, with a prevalence of 41.9% among all adults [1]. In particular, obesity poses several risks and consequences for patients contributing to chronic kidney disease (CKD) and eventually end-stage renal disease (ESRD) [2–4]. In the U.S., the prevalence of CKD among adults is just under 15%, while the prevalence of ESRD remains among one of the highest in the world, with 2,242 cases per million population in 2018 [5]. Obesity prevalence among kidney transplant patients is even higher that then general population at 60% [6], and leads to an increased risk of complications across all stages of care including pre-, peri- and post-operatively. For patients on the kidney transplant wait list, obesity increases the risk of morbidity and metabolic disturbances, and is associated with longer wait times [2, 7, 8]. Moreover, obesity has implications peri- and post-transplant with increased incidence of wound complications, prolonged length of stay, increased morbidity, and delayed graft function [2, 7–9].

Current strategies to address weight loss for kidney transplant patients include lifestyle modifications (diet and exercise), bariatric surgery, and pharmacotherapy [3, 10]. For patients with a body mass index (BMI) > 35, transplant nutrition consult and lifestyle modifications are recommended [3, 10]. However, pre-kidney transplant patient perspectives of these interventions, perceptions of their own weight change, and their willingness and motivations to lose weight, is not well understood.

We sought to identify pre-kidney patient perspectives on weight, weight loss or weight maintenance motivations, resources used for weight loss, and barriers to reaching their weight goal. Understanding patient perspectives of their weight and motivations to lose weight will provide a basis for designing an effective patient-centered weight management protocol for those on the kidney transplant waiting list.

## Methods

A survey was distributed via email to patients on the kidney transplant waitlist at Massachusetts General Hospital. The survey was open for completion for 2 months. Automated reminders to complete the survey were sent through REDCap every 2 weeks. This study was approved by the Mass General Brigham IRB as an exempt study, number 2020P003378.

### Inclusion Criteria

Patients who were 18 years and older on the kidney transplant waitlist were eligible to participate in the study.

### Exclusion Criteria

At the time of data analysis, patients whose survey responses could not be linked by email address to their demographic data, were not included in the study. There were 17 patients who did not have email addresses associated with their survey responses and hence, were excluded. Among non-responders, 71 patients with incomplete data were not included.

### Survey Development

A quantitative and qualitative survey was developed by a multidisciplinary team of healthcare professionals including members of the transplant team, dieticians, and an obesity medicine physician. The survey was designed to address patients’ perceptions of weight and motivation to lose weight, factors contributing to weight change while on the waitlist, barriers hindering weight loss, interest in and utilization of medical and non-medical weight loss resources, and impact of COVID-19 on weight and physical activity. The survey was tested by research personnel prior to administration.

### Data Collection

Automated reminders to complete the survey were sent through REDCap every 2 weeks. Clinical data including weight, height, most recent BMI and BMI at evaluation, days on the waitlist, current medications, organ transplant status and history of previous transplant, etiology of kidney disease, as well as demographic data including race, gender, and age were collected from each patient’s electronic medical record. The survey results were matched to their demographic and clinical data by email address.

### Data Analysis

Statistical analyses were conducted with StataMP14.0, College Station, Texas. Patients were divided into three groups according to their body mass index (BMI): 1) Normal weight: ≤ 24.9 kg/m^2^; 2) overweight: 24.9–30 kg/m^2^; 3) obese: ≥ 30 kg/m^2^. Differences between groups were tested using Mann-Whitney U test and logistic regression analyses for continuous variables. Categorical variables were evaluated utilizing Fisher’s exact or Pearson’s chi-squared tests as appropriate. Probabilities of less than 0.05 were accepted as significant. Free-text responses were reviewed and grouped according to recurring themes and summarized with descriptive statistics.

### Current Transplant Center Practice

Patients on the kidney transplant waitlist undergo an initial transplant evaluation, during which a comprehensive nutrition assessment is conducted. This assessment includes a review of their medical history, current medications, laboratory results, dietary intake, diet history, physical activity level, frailty assessment, and weight history. If patients have a BMI >38 kg/m^2^, they are provided with guidance based on their responses to the nutrition assessment to help them achieve a weight loss goal of 5% within 6 months. For weight loss education, these patients receive informative pamphlets that define and explain BMI calculation, discuss why weight loss and achieving a lower BMI are recommended for transplantation, and include information about the Mass General Hospital Weight Center, including details on how to initiate their weight loss journey.

Interaction with the nutrition team is generally limited for patients on the waitlist unless weight loss is necessary for them to meet the criteria for activation on the wait-list. In such cases, patients are periodically contacted for support and guidance by the transplant dietician. During the readiness visit, which occurs when transplant is estimated to occur within the next 1 year based on waiting-time or sensitization, a follow-up nutrition assessment is conducted, and patients receive guidance on dietary protocols to follow after transplant.

## Results

Among 1,055 patients who were emailed to participate in the survey, 291 patients responded and could be matched with corresponding demographic data (27.6%). There was a significant different in age, sex, and race among included survey responders versus those invited to participate who did not complete the survey. Participants were more likely to be older, male and white (all *p* ≤ 0.01). There were no significant differences in mean BMI (*p* = 0.2), mean days on the waitlist (*p* = 0.7), or etiology of ESRD (*p* = 0.1) (Table 1).

**TABLE 1:** Demographics.

	Responders (%)	Non-responders (%)	*p*-value
All patients (N)	291	676	
Age, *mean (SD)*	59.6 (12.4)	56.6 (13.3)	*<0.01*
Sex, *n (%)*			*<0.01*
Male	186 (64)	413 (61)	
Female	105 (36)	263 (39)	
Race/Ethnicity, *n (%)*			*0.01*
White	230 (79)	447 (66)	
Asian	20 (7)	54 (8)	
Hispanic/Latino			
Black or African American	22 (8)	106 (16)	
American Indian/Alaska Native	1 (0)	4 (1)	
Other/Declined/Unavailable	18 (6)	65 (10)	
BMI, *mean (SD)*	28.6 (5.5)	29.1 (6.0)	0.21
BMI <24.9 kg/m^2 *n (%)*	74 (25.5)	178 (26.3)	
BMI = 25–29.9 kg/m^2 *n (%)*	112 (38.5)	235 (34.8)	
BMI >30 kg/m^2 *n (%)*	105 (36.1)	263 (38.9)	
Days on waitlist, *mean (SD)*	1,071.4 (2,207.8)	1,118.5 (1,361.5)	0.74
Etiology of Kidney Disease (Primary and Secondary Diagnosis), *n (%)*			0.85
Glomerular Diseases	25 (8)	60 (9)	
Diabetes Nephropathy	81 (25)	203 (29)	
Hypertension	62 (19)	115 (16)	
Polycystic kidney disease	23 (7)	60 (9)	
Congenital	2 (1)	26 (4)	
IgA nephropathy	25 (8)	54 (8)	
Kidney Toxicity + AKI	30 (9)	32 (5)	
Unknown/Other*	75 (23)	149 (21)	

### Perception of Weight and Motivation for Weight Loss

**FIGURE 1:**
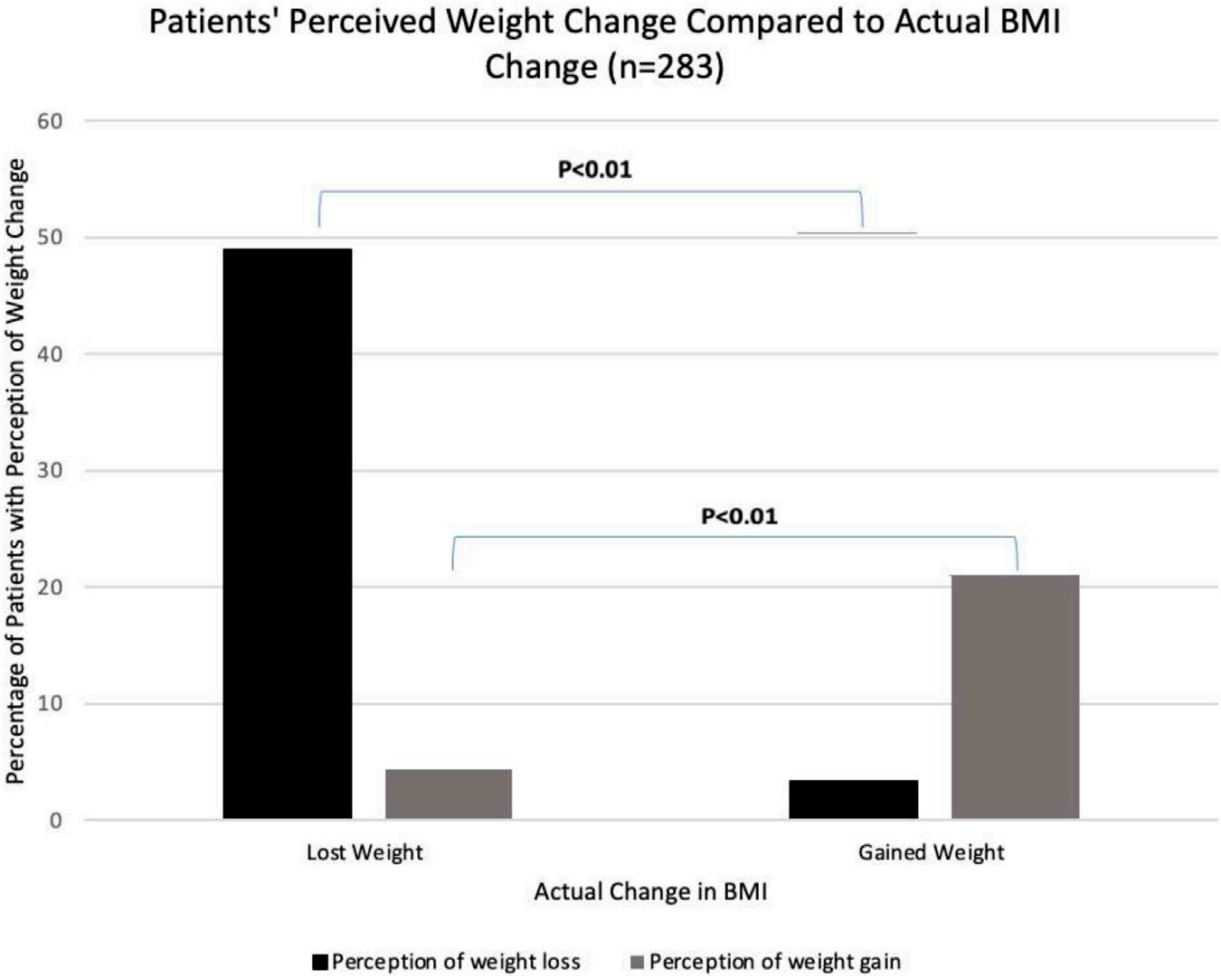
Perception of weight change while on the waitlist was stratified by actual weight change while on the waitlist. The percentage of patients with perception of weight gain or loss (y-axis) accurately reflected actual change in BMI (x-axis) (*p* < 0.01).

Among all patients, actual weight change was correlated with patient’s perception of weight change while being on the waitlist (*p* < 0.01, Figure 1). While 24.91% (*n* = 70) of patients expressed weighing less than what they described as their normal baseline, 14.59% (*n* = 41) expressed weighing more than their normal baseline. Among all survey respondents, 47.1% self-reported that they had lost weight since being waitlisted, whereas 32.6% reported gaining weight since being waitlisted. Of patients who noted weight gain since being waitlisted, 75% (*n* = 21) and 36% (*n* = 10) were seriously considering weight loss and weight maintenance in comparison to those who reported weight loss, no change in weight, or weight fluctuation (*p* < 0.001). Patients with a most recent BMI ≥ 30 kg/m^2^ were more likely to try to not gain weight, seriously consider weight loss, and less likely to seek weight maintenance in comparison to patients with a BMI<30 kg/m^2^ (*p* < 0.01). Further, patients with a BMI ≥ 30 kg/m^2^ were less likely to attribute their weight to their kidney disease compared to normal and overweight (*p* = 0.042).

### Factors Contributing to Weight Change While on the Waitlist

One hundred seventy-eight (66.4%) patients stated they received nutrition counseling prior to responding to the survey. Receiving nutrition counseling was significantly associated with an increase in daily servings of vegetables consumed (*p* = 0.024) and a significantly reduced number of eating meals out (*p* = 0.041). Nutrition counseling was not significantly associated with BMI or weight change.

### Barriers for Weight Loss

**FIGURE 2:**
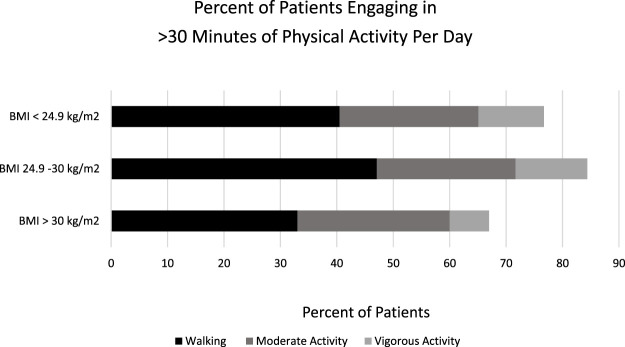
Engagement in physical activity was quantified and stratified by BMI. Individuals categorized as “overweight” demonstrated the highest engagement, with more than 30 minutes of physical activity.

Among patients with a BMI exceeding 25 kg/m^2^, 53.5% identified experiencing barriers to weight loss. Among all respondents, regardless of BMI, 10.3% reported difficulty in maintaining a specific or restrictive diet, while 12.4% mentioned intolerance or a reduced ability to engage in physical activity, often associated with fatigue symptoms. Furthermore, 15.1% of patients cited comorbidities and treatment side effects as factors hindering their ability to lose weight (Figure 2).

### Interest and Utilization of Medical and Non-Medical Resources

Patients most frequently listed utilization of dietary modifications to maintain weight (68%, *n* = 198) followed by using home exercise programs (39%, *n* = 113), calorie tracking (20%, *n* = 59), other strategies most commonly including aerobic activity and dietary changes (20%, *n* = 58), a gym membership (13%, *n* = 38), and a personal trainer (3%, *n* = 9) (Table 2). When asked about the potential future use of weight loss or maintenance strategies, patients were equally interested in utilizing home exercise programs or a personal trainer (23%, *n* = 68), followed by a gym membership (21%, *n* = 62), calorie tracking (15%, *n* = 44), dietary modifications (12%, *n* = 36), and others (8%, *n* = 22).

**TABLE 2:** Current and future utilization of weight management resources.

Weight management strategies	Current use			Future use		
	N (%)	Or (95% CI)	*p*-value	N (%)	Or (95% CI)	*p*-value
Dietary Modifications	198 (68)	1.17 (0.85–1.60)	0.34	36 (12)	1.32 (0.83–2.10)	0.24
Home Exercise Program	113 (39)	0.87 (0.64–1.17)	0.35	68 (23)	1.17 (0.82–1.66)	0.40
Calorie Tracking	59 (20)	1.37 (0.94–1.99)	0.14	44 (15)	1.62 (1.04–2.51)	0.03
Others	58 (20)	0.96 (0.66–1.39)	0.82	22 (8)	0.97 (0.56–1.70)	0.92
Gym Membership	38 (13)	1.7 (1.06–2.72)	***0.03***	62 (21)	0.98 (0.68–1.41)	0.91
Personal Trainer	9 (3)	1.88 (0.72–4.91)	0.20	68 (23)	1.20 (0.85–1.71)	0.31

Weight management devices	N (%)	Or (95% CI)		N (%)	Or (95% CI)	*p*-value
I don’t wish to track my weight	161 (55)	0.85 (0.63–1.14)	0.28	100 (34)	0.75 (0.55–1.02)	0.07
Smart Phone	52 (18)	0.98 (0.67–1.44)	0.92	42 (14)	1.51 (0.97–2.34)	0.07
Smart Watch	49 (17)	1.17 (0.78–1.74)	0.45	52 (18)	0.81 (0.55–1.19)	0.28
Fitbit	28 (10)	0.94 (0.57–1.55)	0.80	48 (16)	1.46 (0.97–2.21)	0.07
Others	13 (4)	1.67 (0.77–3.64)	0.19	12 (4)	1.29 (0.60–2.77)	0.52
Social Media Fitness App	9 (3)	2.44 (0.86–6.90)	0.09	30 (10)	1.36 (0.82–2.24)	0.24
Pedometer	9 (3)	1.01 (0.43–4.37)	0.99	45 (15)	1.32 (0.87–2.01)	0.20
GPS Enabled Watch	5 (2)	1.17 (0.37–3.73)	0.79	21 (7)	1.27 (0.71–2.28)	0.42

When stratified by BMI, patients with a higher BMI were significantly more likely to utilize a gym membership (*p* = 0.028). In comparison to participants with a BMI ≤24.9 kg/m^2^ participants with a BMI 24.9–30 kg/m^2^ had a odds ratio of 0.026, (95% CI: 0.073–0.125), and participants with a BMI ≥ 30 kg/m^2^ had an odds ratio of 0.109 (95% CI 0.009–0.21). Also, regarding future weight loss strategies, participants with a BMI 24.9–30 kg/m^2^ and a BMI ≥ 30 kg/m^2^ were more interested (*p* = 0.032) in calorie tracking in in comparison to patients with a BMI ≤ 24.9 kg/m^2^. Otherwise, there was no difference in patient’s future strategies for weight loss/maintenance based on BMI category.

When discussing weight management devices, the majority of patients indicated that they do not currently track their weight (55%, *n* = 161). Among those who currently track their weight, the most common method of weight tracking was a smart phone (18%, *n* = 52). This was followed by a smart watch (17%, *n* = 49), Fitbit (10%, *n* = 28), social media fitness application or a pedometer (3%, n = 9), a GPS enabled watch (2%, *n* = 5), and others (4%, *n* = 13). While most patients (34%, *n* = 100) selected that they are not interested in tracking their weight in the future, 18% (*n* = 52) expressed interest in a smart watch, followed by a Fitbit (16%, *n* = 48), pedometer (15%, *n* = 45), smartphone (14%, *n* = 42), GPS enabled watch (7%, *n* = 21), and others (4%, *n* = 12) (Table 2). There were no significant differences in current weight management device utilization or interest in future utilization of weight management resources when stratified by BMI.

### Interest in Weight Center Resources and Medically Assisted Weight Loss

While the majority of patients were not interested in a referral to the weight center, weight loss medication, or weight surgery (71%, *n* = 206), there was a significant correlation between higher BMI and patient interest in these resources (Table 3). While weight loss surgery was of least interest to patients with a BMI ≥ 30 kg/m^2^ (11%, *n* = 12), it was appropriately significantly greater among higher BMI patients in comparison to normal or overweight.

**TABLE 3:** Patient interest in medical resources for weight loss.

	**BMI N (%)**			**Or (95% CI)**	***p*-value**
	**<24.9**	**24.9–29.99**	**>30**		
**Weight center referral**	4 (5)	12 (11)	28 (27)	2.68 (1.63–4.42)	*<0.01*
**Weight loss medication**	0 (0)	6 (5)	28 (27)	7.79 (3.35–18.13)	*<0.01*
**Weight loss surgery**	1 (1)	1 (1)	12 (11)	5.48 (1.72–17.50)	*0.01*
**Not interested**	65 (88)	87 (78)	54 (51)	0.36 (0.25–0.53)	*<0.01*

### Physical Activity and Impact of COVID-19

There were no significant differences in physical activity as identified by engaging in walking, moderate exercise, or vigorous exercise among patients when stratified by BMI (Figure 2). Among all patients, more patients engaged in less than 30 min of physical activity than more than 30 min of activity.

Participants were asked about both activity change and weight change during the COVID-19 pandemic. Regarding activity during COVID, 46.1% reported decreased activity during the pandemic, whereas 43.1% reported no change in physical activity. Furthermore, patient reporting of activity change during COVID-19 did not differ among patients when stratified by BMI (*p* = 0.548).

While the majority of patients, regardless of BMI, indicated no COVID-associated weight change, overall patients were more likely to indicate weight loss than gain during COVID-19. Additionally, there was a significant difference in weight change during the pandemic when stratified by BMI (*p* = 0.025). Among those who indicated more than 10 pounds of weight loss, patients with BMI ≥ 30 kg/m^2^ had the highest proportion of weight loss during COVID-19, followed by patients with a BMI between 24.9–30 kg/m^2^, and patients with BMI. Similarly, a higher proportion of obese patients lost <10 pounds, in comparison to overweight and normal weight patients. Among those who indicated weight gain greater than and less than 10 pounds, there was a higher proportion of patients with a BMI ≥ 30 kg/m^2^ in comparison to those BMI 24.9–29.9 kg/m^2^ and BMI ≤ 24.9 kg/m^2^.

## Discussion

This study surveyed patients currently on the kidney transplant waitlist at a single institution and examined their interest in and utilization of resources for weight maintenance, physical activity, perception and understanding of weight, and motivation to lose weight. Patients with a higher BMI expressed greater interest in use of weight loss resources in the future, such as utilizing a gym membership (*p* = 0.028), and calorie tracking (*p* = 0.104). Patients with a higher BMI were more likely to express openness to weight center referral (*p* < 0.001), weight loss medication (*p* < 0.001), and weight loss surgery (*p* = 0.004). Nutrition counseling was associated with a significant increase in vegetable consumption (*p* = 0.024) but no difference in BMI or weight loss. There were no significant differences in physical activity when stratified by BMI or impact of COVID-19. Obese patients, however, expressed greater fluctuation in weight during the pandemic (both weight loss and weight gain).

Regarding actual weigh change, over time, patient reported weight change accurately reflected weight change by change in BMI at listing and most recent (*p* < 0.01). Patients with a higher BMI were more motivated to lose weight and try not to gain weight (*p* < 0.01). Such findings suggest that patients whose weight is of greatest concern prior to kidney transplant are most interested in seeking resources for weight loss, both medical/surgical and non-medical, and motivated to lose weight. At our single center study, nutrition counseling was associated with an increase in healthy dietary behaviors, as defined as vegetable consumption and not eating out.

As obesity rates increase and result in reduced kidney graft survival and increased patient complications [11], methods to educate patients and help manage weight loss in the context of transplant surgery is of increasing significance. Approximately 60% of patients undergoing a kidney transplant have a BMI>30 kg/m^2^ with frequent additional weight gain post-transplant [12]. One study demonstrated that patients with a BMI>30 kg/m^2^ experience longer procedure times and warm ischemia, though there was no difference associated with BMI 24.9–30 kg/m^2^. Furthermore, kidney graft function post-operatively was reduced after a 1-month among patients who were BMI>30 kg/m^2^ [11].

Many transplant centers are adjusting for the increase in obesity and raising the limits of accepted pre-transplant BMI, but ongoing work to improve the safety and efficacy for obese patients receiving a kidney transplant is still underway [13]. According to the Kidney Disease Improving Global Outcomes (KDIGO) Clinical Practice Guidelines, patients should not be not excluded from transplantation due to BMI or waist-to-hip ratio status, but patients with obesity should be offered weight loss interventions prior to transplantation [14]. The American Society of Transplantation noted that low-calorie diet, behavioral therapy, and a physical activity program to achieve a BMI<30 kg/m^2^ is recommended as a goal for pre-transplant patients [12].

Regarding weight loss strategies, Yemini et al., demonstrated successful bariatric surgery prior to kidney transplant for morbidly obese patients resulting in reduced obesity related comorbidities peri-transplant [12]. Kukla et al., performed a clinical cohort study that compared weight loss pre-kidney transplant for patients with diabetes receiving a conservative approach to weight loss including individualized nutrition and physical activity regimens as well as specialist consultations, in comparison to patients who received bariatric surgery [15]. It was found that patients who opted for the conservative approach lost 3% of their body weight at 1-year post-weight consultation, whereas patients who underwent a bariatric surgery lost 19% of their body weight [15].

In our study, patients who were at greatest risk for kidney transplant complications (BMI ≥ 30), were most motivated to lose weight and receive resources, both through support from the weight center and personal fitness devices and activities. Given that weight can be a barrier to receiving a transplant and impacts patient and graft survival after transplant, further work is needed to address access to and utilization of weight loss resources and education, as well as to understand how self-perception and cultural values may impact weight loss for pre-kidney transplant patients.

### Limitations

There are noteworthy limitations to be mentioned regarding the generalizability of the study findings. First, the primary method of stratifying patients was by BMI, which does not differentiate tissue type and fluid retention. It is recommended that waist circumference be used as an additional method to measure abdominal adiposity, but this was not feasible as it is not routinely collected in our transplant center and therefore is not available from chart review [16]. Additionally, we did not have patients self-report their weight. Weight and BMI were attained from the medical record. With the increase in telehealth visits due to the COVID pandemic, the patients may have been in clinic less frequently which may affect the accuracy of the recorded weight in the medical record. Second, deconditioning because of dialysis and kidney disease may limit pre-transplant patients’ physical activity and increase weight fluctuation. While frailty scores are currently collected at our evaluation clinic, this is a change in practice, and they are not available from the chart review we performed.

Third, by nature of this survey study, limited email or internet access may affect response rate, and participation bias may impact the results. Fourth, the data presented is from a single center, and there were statistically significant differences in some demographic features between respondents and non-respondents, limiting the generalizability of the findings. Fifth, we chose to not include questions assessing patients’ knowledge of their disease and healthy weight in order to keep the survey brief to optimize response rates; however, further studies could assess patient knowledge as that may impact their behavior regarding weight loss and weight maintenance. Despite these limitations, the findings demonstrate valuable insights regarding the patient’s perspective on pre-kidney transplant weight reduction, interest in weight loss resources, impact of weight center interventions on health behaviors, and perception of weight change that reflects weight maintenance motivation.

## Conclusion

To our knowledge, this is the first study published on patients’ perspectives and willingness to lose weight while on the kidney transplant wait list. The findings from this survey will be the basis of the development of focus group guides to further explore patient perceptions of pre-transplant weight loss. Through this research and the planned future studies, weight management protocols may be optimized to best address the current increasing trend of obesity in -pre kidney transplant patients.

## Data Availability

The raw data supporting the conclusion of this article will be made available by the authors, upon completion of a formal Data Use Agreement process.

## Abbreviations

CKD: chronic kidney disease; ESRD: end stage renal disease; BMI, body mass index.
